# Limited Population Structure, Genetic Drift and Bottlenecks Characterise an Endangered Bird Species in a Dynamic, Fire-Prone Ecosystem

**DOI:** 10.1371/journal.pone.0059732

**Published:** 2013-04-23

**Authors:** Sarah M. Brown, Katherine A. Harrisson, Rohan H. Clarke, Andrew F. Bennett, Paul Sunnucks

**Affiliations:** 1 School of Life and Environmental Sciences, Deakin University, Burwood, Australia; 2 School of Biological Sciences, Monash University, Clayton, Australia; The Australian National University, Australia

## Abstract

Fire is a major disturbance process in many ecosystems world-wide, resulting in spatially and temporally dynamic landscapes. For populations occupying such environments, fire-induced landscape change is likely to influence population processes, and genetic patterns and structure among populations. The Mallee Emu-wren *Stipiturus mallee* is an endangered passerine whose global distribution is confined to fire-prone, semi-arid mallee shrublands in south-eastern Australia. This species, with poor capacity for dispersal, has undergone a precipitous reduction in distribution and numbers in recent decades. We used genetic analyses of 11 length-variable, nuclear loci to examine population structure and processes within this species, across its global range. Populations of the Mallee Emu-wren exhibited a low to moderate level of genetic diversity, and evidence of bottlenecks and genetic drift. Bayesian clustering methods revealed weak genetic population structure across the species' range. The direct effects of large fires, together with associated changes in the spatial and temporal patterns of suitable habitat, have the potential to cause population bottlenecks, serial local extinctions and subsequent recolonisation, all of which may interact to erode and homogenise genetic diversity in this species. Movement among temporally and spatially shifting habitat, appears to maintain long-term genetic connectivity. A plausible explanation for the observed genetic patterns is that, following extensive fires, recolonisation exceeds *in-situ* survival as the primary driver of population recovery in this species. These findings suggest that dynamic, fire-dominated landscapes can drive genetic homogenisation of populations of species with low-mobility and specialised habitat that otherwise would be expected to show strongly structured populations. Such effects must be considered when formulating management actions to conserve species in fire-prone systems.

## Introduction

Fire is a major disturbance process that changes landscape structure in many ecosystems worldwide [1], [2] and has profound impacts on biodiversity [3]. Substantial changes in species diversity and community structure may result from fire, including an increased risk of extinction for populations [4]–[6]. Fires initiate spatial and temporal changes in resources, which alter the suitability of habitat for species [7]. This can lead to patchily distributed populations with consequences for population demography [8], [9], genetic structure [10]–[12] and metapopulation dynamics [13].

The effects of fire-induced landscape change on ecological and population processes are complex, and major gaps remain in our knowledge [7], [14], [15]. Fire directly reduces population size [8], [9], [16] and in severe events may cause temporal bottlenecks in population size. Bottlenecks often drive a loss of genetic diversity and inbreeding that may lead to the accumulation and expression of deleterious alleles, inbreeding depression and the subsequent reduction of population viability [17]–[19]. Changes in the spatial pattern of habitat resulting from fire can also increase the isolation of populations [20] and alter the movement of animals between patches [21], [22].

Following disturbance by fire, subsequent population recovery will be influenced by many factors including the number and demographic parameters of survivors [8], [23], resource availability [24], post-disturbance successional pathways [7], [25], species' dispersal ability [26] and the geographic scale and patchiness at which the fire occurred [7], [9], [27]. Understanding the consequences of disturbance by fire for population processes is profoundly important for the management of species in fire-prone environments. This includes the need to develop appropriate fire regimes [14], [16], [23], to assess the susceptibility of populations to extinction [28] and to mitigate potentially adverse effects of post-fire disturbance activities [9].

**Figure 1:**
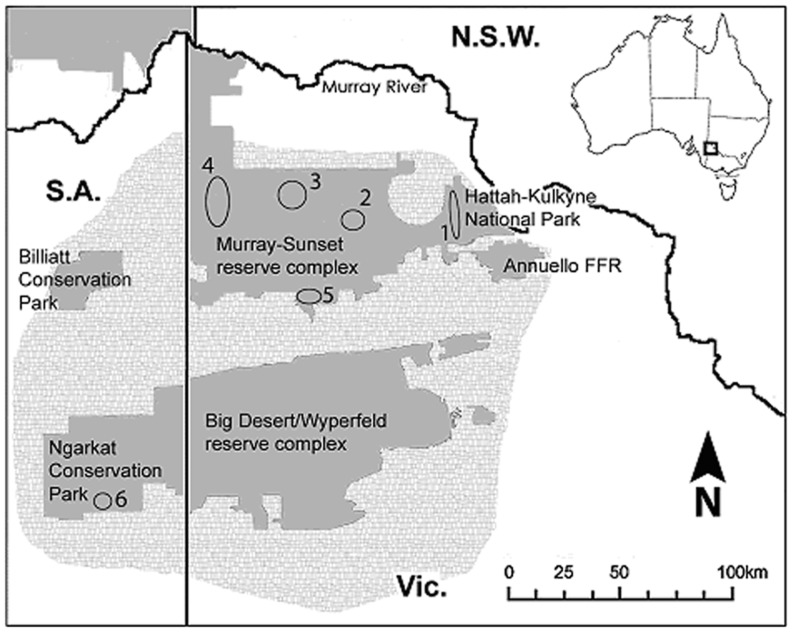
Map of sampling localities within the conservation reserve system of the Murray Mallee region, south-east Australia. Numbered ellipses indicate sites where Mallee Emu-wrens were sampled. 1 Hattah-Kulkyne National Park (NP), 2 Murray-Sunset NP (East), 3 Murray-Sunset NP (Central), 4 Murray Sunset NP (West), 5 Murray Sunset NP (South) and 6 Ngarkat Conservation Park. The historic distribution is represented by light grey stippling (Higgins et al. 2001). Solid grey represents the reserve system in Victoria and South Australia. FFR  =  Fauna and Flora Reserve. New South Wales (N.S.W.), South Australia (S.A.) and Victoria (Vic.).

This study examines the genetic diversity and structure of a globally endangered bird species, the Mallee Emu-wren *Stipiturus mallee*. The Mallee Emu-wren is one of the tiniest members (4–6.5 g) of the family Maluridae, endemic to the semi-arid zone of south-east Australia [20], [29], [30] (Figure 1). This zone encompasses extensive tracts of 'mallee' shrublands dominated by 3–10 m tall *Eucalyptus* spp. ‘Mallee’ refers to the growth form of *Eucalyptus* spp. characterised by a large underground lignotuber from which multiple stems sprout. The Mallee Emu-wren is a resident habitat specialist and has a patchy distribution. Multi-scale studies of the habitat requirements of this species show that its distribution is strongly influenced by the post-fire age of vegetation, as a consequence of seral changes in the structure of ground-storey spinifex grass (*Triodia scariosa*) on which it depends [20], [31]. In the northern area of its distribution (i.e. Murray-Sunset and Hattah-Kulkyne National Parks), it inhabits *Triodia-*mallee vegetation older than 15 years since last burnt, with a slight preference for vegetation 15–29 years of age [20]). There are no studies on dispersal of this inconspicuous species. Nonetheless, its short, rounded wings and long filamentous tail, which allow it to scurry through dense spinifex grass, make it a notoriously poor flier likely to have limited dispersal capability [30].

In recent decades, the Mallee Emu-wren has undergone a precipitous reduction in numbers and distribution. Large wildfires (>10 000 ha), exacerbated by drought, have caused the extinction of populations in parts of the south-west of its range. Large fires and inappropriate fire regimes that reduce the amount of habitat of suitable age are considered a significant threat to remaining populations. Geographic range contraction has occurred such that the Murray-Sunset and Hattah-Kulkyne National Parks in northern Victoria now support an estimated 92% of the global population. Small, scattered and isolated populations occur in South Australia in Billiatt and Ngarkat Conservation Parks and only one pair was recorded in the Big Desert/Wyperfeld reserve complex in Victoria in 2006 [20]. Extensive expert survey in 2006 of areas that once held the species (Wathe and Bronzewing Fauna and Flora Reserves) failed to find any Mallee Emu-wrens [20](Figure 1).

This study examines the genetic diversity and structure of the Mallee Emu-wren across its global range. The primary objective was to examine spatial genetic patterns among populations across the species' range to draw inferences about population structure and processes. Its occurence in a fire-prone environment, coupled with the intrinsic characteristics of the Mallee Emu-wren (e.g. poor flight capability) strongly suggest that it may exhibit a metapopulation structure at the landscape-scale, with relatively strong patterns of genetic divergence (i.e. population genetic structure) expected among geographically-dispersed sampling units [32]. Such insight would be important for the management of this species, including the potential need for relocation and population augmentation.

## Materials and Methods

### Study area and sample collection

The Mallee Emu-wren is inconspicuous and rare, has patchy distribution and occur in low density [20], making it difficult to locate and capture. Samples were collected during 2006–2008 as part of a wider study of the ecology of this species. Blood and feather samples were analysed from 72 individuals from six locations across the global range of the Mallee Emu-wren in south-eastern Australia (bounded by 34°50′S–36°00′S, 140°00′N–142°50′N). Four locations were within the Murray-Sunset National Park (n = 10, 10, 12, and 6). The other locations sampled were Hattah-Kulkyne National Park (n = 28) and Ngarkat Conservation Park (n = 6) (Figure 1, Table 1). Based on contemporary survey estimates, the proportion of the population sampled from the Murray-Sunset National Park is less than 1%. The proportion of the population sampled from Hattah-Kulkyne National Park is about 10% [20], and 15% of the known population of Ngarkat Conservation Park was sampled in 2007 (i.e. six of 20 male/female pairs; C. Hedger, personal communication). Individuals sampled within the Murray-Sunset and Hattah-Kulkyne National Parks, were obtained from vegetation greater than 17 years since last burnt. The samples from Ngarkat Conservation Park originated from mid to late-age mallee-heath vegetation of similar structure (exact age unknown).

**Table 1:** Sample size (n), allelic richness (AR), observed (Ho) and expected heterozygosity (UHe), and inbreeding coefficient (*F*_IS_) for 72 individuals of the Mallee Emu-wren from six locations across the species' global range.

Population	n	Males	Females	AR	Ho	UHe	*F*_IS_	H-W disequilibrium	Monomorphic loci
Hattah-Kulkyne NP	28	15	13	3.57	0.41	0.46	0.124*	Smm1, Smm3	Msp6, Smm6, Smm7
Murray Sunset NP (East)	10	5	5	3.06	0.46	0.45	−0.038		Mcy7, Msp6, Smm6, Smm7
Murray Sunset NP (Central)	10	5	5	3.83	0.44	0.49	0.095	Mcym4	Smm6, Smm7
Murray Sunset NP (West)	12	7	5	3.80	0.53	0.52	−0.022		
Murray Sunset NP (South)	6	4	2	3.50	0.43	0.47	0.094		Msp6, Smm6, Smm7
Ngarkat Conservation Park	6	2	3	2.92	0.43	0.43	−0.094		15144s1, Mcy7, Msp6, Smm6, Smm7

* *p* = 0.05.

Long-term ecological and genetic studies on other species of the family Maluridae show males are philopatric, and related males (e.g. brothers) have a tendency to occupy neighbouring territories. Dispersal tends to be female biased, although the pattern and distance varies within populations and among species [30]. A preliminary population study of the Mallee Emu-wren, in which individuals were individually banded, showed that males occupied overlapping breeding territories of about 5 ha (S. Brown, unpublished data). On this basis, samples for population analyses were collected at minimum intervals of 500 m, and where possible, at about 2 km intervals, to minimise potential non-random sampling (i.e. sampling related individuals). However, because this species is rare and has a highly patchy distribution, this was not always possible. With the exception of Hattah-Kulkyne National Park, locations were visited only once, hence avoiding temporal sampling of offspring. Due to severe drought in south-eastern Australia (from 1997 up until the period of sampling, 2006–08 [33]), breeding was uncommon (S. Brown, personal observations) and consequently most samples collected comprised only of a male/female pair from any given site. Parentage analysis (CERVUS 3.0, [34], Text S1, Table S1) was used to identify possible parent-offspring pairs among individuals. Six potential offspring originating from Hattah-Kulkyne National Park were removed from analysis (Text S1, Table S1). Known offspring and individuals that had more than 2 loci missing were excluded (in total, n = 15 were excluded from 87 original samples).

Mallee Emu-wrens were captured either by trapping in monofilament mist nets, or by throw nets after being lured by playback recordings of calls (©David Stewart/Nature Sound). The species is sexually dichromatic and males are easily distinguished from females by the presence of brilliant sky-blue throat and breast feathers [30]. Between 10 and 50 µl of blood was collected from the brachial vein, or a single pin feather was removed, and material stored in 70% molecular grade alcohol, for subsequent extraction of DNA and genetic analyses.

### Ethics Statement

All work was conducted under Deakin University Animal Ethics Committee approval (A35/2005) and research permits issued by the Department of Sustainability and Environment, Victoria (10003389) and the Department of Environment and Heritage, South Australia (G24995, 29/2005-M1).

### Molecular marker selection and PCR

DNA was extracted from samples using a standard ethanol/chloroform extraction method [35]. Samples were genotyped at 12 variable nuclear loci. Eleven microsatellites were amplified using primers developed for the Splendid Fairy-wren *Malurus splendens* [36] Superb Fairy-wren *M. cyaneus* [37] and Southern Emu-wren *Stipiturus malachurus* [38]. A single Exon-Primed-Intron-Crossing (EPIC) region was amplified [39], with primers re-designed so that the product was of a suitable length to be run on a Li-Cor 4300 Global IR2 two-dye DNA sequencer. IRD-labelled M13 primer was added at 0.1 µM to the EPIC PCR reactions, so that alleles could be visualized by electrophoresis on 6% polyacrylamide sequencing gels. Four of the six microsatellite primer pairs from the Southern Emu-wren were re-designed so that two panels of microsatellite products could be run on an ABI® capillary system (Table 2).

**Table 2:** Hypervariable-length nuclear loci used in this study and their characteristics.

	Locus	Reference	Accession N^O^	5′ Primer	3′ Primer	*bp* #	N*_A_*
**Microsatellites**	Smm1†	[36]	DQ160181	TGGGAATGCTCTATTTCTGG	ACTCCATGGAACTCCAGACG	274–330	15
	Smm2		DQ160185	CCAAGACCTGACACTTACGC	CACAGAGGAGCTCACACACG	203–398	26
	Smm3		DQ160186	CATATGAATGTAGCAGCTGGG	CATGGCACAGTGAGCTGG	299–497	32
	Smm5†		DQ160184	TCAGGGAGAAAAAGCAAGGA	CCCTGAGTGACCCTGATGTT	309–351	3
	Smm6†		DQ160183	AAAGCTGCGTATCCCAAGG	GCAAATCTGGTGAGCTGTGA	441–443	2
	Smm7†		DQ160182	TGCTCTGGTTTGACTGATGC	GCCAGCCAGGATGCTATTTA	187–189	2
	Mcy7	[35]	U82391	CTTTGTGTTGCTGTTAGGTAGAA	GGCTCAACAGCTATTTGCAT	86–88	2
	Mcy4		U82388	ATAAGATGACTAAGGTCTCTGGTG	GGCTCAACAGCTATTTGCAT	158–180	10
	Msp4		AY320050	GGAGAGACCGGGAAACAGAGAC	′TAGCAATTGTCTATCATGGTTTG	167–174	3
	Msp6		AY320051	GCAGGTTTTTAATGGCATCAAG	GCAGGTTTTTAATGGCATCAAG	237–241	2
	Msp10		AY320051	CGCGTCAAATAAGGGGGAAACC	CGCGTCAAATAAGGGGGAAACC	143–173	9
**EPIC**	15144s1§	[37], [71]	P23913	TTGAACCCTCGTATTGGCAG	ATGGTTTTCATTTGCCMCAA	292–294	2

# Microsatellite sizes detected in the Mallee Emu-wren.

† Primers re-designed from Genbank submission sequence clones [38].

§ Primers modified from Backström *et al.* [39], [81].

*bp* = allele size range, N*_A_* = number of alleles.

PCR reactions for microsatellites and product separation were performed in two different laboratories, with cross-referencing quality control. PCR reactions for each marker were optimised using the following: 20–40 ng of sample DNA, 0.5 Units Go *Taq* DNA polyermase, 5× buffer, 0.25 mM dNTPs, 0.5–4.5 mM MgCl_2_ (Promega/MBI Fementas) and 5–10 pmol of each primer pair in a total volume of 20 µl. Microsatellite products were run on an ABI® Capillary Analyser (Perkin Elmer) or a Li-Cor 4200 and 4300 Global IR2 two-dye DNA sequencer for separation and sizing. Putative homozygotes were amplified and genotyped twice to confirm their status. Positive and negative controls were used in all reactions.

### Genetic diversity

Standard measures of genetic diversity for length-variable markers were obtained in various programs, treating the six geographic locations as separate populations. GENALEX V6.0 [40] was used to calculate observed (Ho) and unbiased expected heterozygosity (UHe). Unbiased He was used as this metric is better suited than standard He for estimating heterozyosity when sample sizes are low [41]. Allelic richness (AR, i.e. allelic diversity corrected for differences in sample size) and inbreeding coefficient (*F_IS_*) were calculated in FSTAT 2.9.3.2 [42]. Tests for deviations from Hardy-Weinberg equilibrium and linkage equilibrium were performed using GENEPOP V4.0 [43].

Enhanced effects of genetic drift in small, isolated populations are expected to lead to increased genetic differentiation among sites. The extent of genetic differentiation between each pair of sites was estimated using two allele-frequency-based measures: *F*_ST_ calculated in GENEPOP V4.0 [43] and Jost's *D*, adjusted for small sample sizes (*D_est_*)[44], calculated in the DEMEtics package in R [45]. *F*_ST_ and *D_est_* as measures of genetic differentiation each have different strengths and shortcomings, so we present both to infer population differentiation [46]. Both *F*_ST_ and *D_est_* values theoretically range from zero to one, with zero indicating no differentiation (i.e. no differences in allele frequencies and one representing maximum differentiation) [41]. *F*_ST_ is a widely used estimator of population genetic structure, with which many researchers are familiar [46], [47]. On the other hand, Jost's *D* may be more appropriate for highly-variable markers (e.g. microsatellites) [44], [48]. Furthermore, simulations have shown that G'*_ST_*, (which is almost perfectly correlated with Jost's D [49], which is used here), accumulates faster than F_ST_ following the introduction of barriers to gene flow [50]. Because *F*_ST_ and Jost's *D* are allele-frequency-based analyses, they should reflect processes operating on longer time-scales than individual, genotype-based analyses (e.g. STRUCTURE, TESS; see below).

### Bottleneck analysis

The heterozygosity excess test in the program BOTTLENECK V1.2.03 [51] was used to ascertain whether recent declines (within several dozen generations) in population size have occurred in the Mallee Emu-wren [52]. Populations that have recently experienced a bottleneck lose relatively more allelic diversity (through loss of rare alleles) than heterozygosity relative to that expected if a population was at mutation-drift equilibrium [52]. This heterozygosity excess should not be confused with that underpinning *F*_IS_ - which is an expression of excess of heterozygotes relative to proportions expected under Hardy-Weinberg equilibrium [46]. The heterozygosity excess test is reasonably robust to incorrect assumptions about mutation models [53]. Significance of heterozygosity excess was determined using the Wilcoxon signed-rank test, as it is robust to the effects of both small samples sizes (<30) and a small number of loci (<20) [51]. Tests for heterozygosity excess were performed using a two-phase mutation model (TPM) in BOTTLENECK, with the proportion of stepwise mutations set to 90%.

### Population structure

To assess the extent of genetic population structure across the species' global range, individual genotype-based Bayesian clustering algorithms were implemented in both STRUCTURE 2.3.3 (without spatial information) [54], [55] and TESS 2.3.1 (incorporating spatial information) [56], [57]. Because these analyses are individual based, TESS and STRUCTURE are less biased by effects of small sample sizes or violations of assumptions of Hardy-Weinberg equilibrium, compared with population based analyses.

Structure was run using the admixture model with correlated allele frequencies, and *K* values 1 to 10. Twenty replicate runs were performed for each *K* value. Each run was 3×10^6^ Markov Chain Monte Carlo (MCMC) repetitions following a burn-in period of 10^6^ repetitions.

TESS incorporates information on individual geographic coordinates and has been shown to be more powerful than non-spatial algorithms, especially in weakly differentiated populations [56]–[58]. TESS was run using the conditional autoregressive model (CAR) admixture model with spatial interaction parameter (i.e. weighting of the geo-coordinates) set at 0.6, as recommended for *K*≈5 populations [56]. One hundred replicate runs of 100 000 sweeps (disregarding the first 30 000) sweeps were performed for *K* values 2 to 9. The Deviance Information Criterion (DIC) was used to select the model that best fit the genetic data [59]. DIC values averaged over 100 independent iterations were plotted against *K*, and the most likely value of *K* was selected by visually assessing the point at which DIC first reached a plateau and the number of clusters to which individuals were proportionally assigned. The 10 runs with the lowest DIC values for the selected *K*-value were retained and their admixture estimates were averaged using CLUMPP V 1.1.2 [60], applying the greedy algorithm with random input order and 1000 permutations to align the runs and calculate G' statistics. Results were visualised using DISTRUCT 1.1 [61].

## Results

### Genetic diversity and bottlenecks

Analyses of the nuclear loci show overall moderate to low levels of genetic diversity across the global range of the Mallee Emu-wren, with signatures of recent population bottlenecks in two locations, and local effects of genetic drift in others such that only a single sampled population showed neither phenomenon (Tables 1, 3 & 4).

**Table 3:** Measures of pairwise differentiation for six location samples of the Mallee Emu-wren based on; i) *F*_ST_ (below the diagonal) and ii) *D_est_* (above the diagonal).

	Population	1	2	3	4	5	6
**1**	Hattah-Kulkyne NP		0.101*	0.044	0.077*	0.035	0.122*
**2**	Murray-Sunset NP (East)	0.037*		0.081*	0.105*	0.103*	0.092*
**3**	Murray-Sunset NP (Central)	0.011*	0.014*		0.004	0.020	0.104*
**4**	Murray Sunset NP (West)	0.020*	0.025*	0.000		0.000	0.132*
**5**	Murray Sunset NP (South)	0.002	0.030*	0.000	0.000		0.179*
**6**	Ngarkat CP	0.027*	0.032*	0.018	0.041*	0.044*	

* *p*<0.05.

**Table 4:** Results from the BOTTLENECK test of microsatellite from the six populations.

Population	TPM
Hattah-Kulkyne NP	0.213
Murray Sunset NP (East)	0.002*
Murray Sunset NP (Central)	0.188
Murray Sunset NP (West)	0.601
Murray Sunset NP (South)	0.285
Ngarkat Conservation Park	0.004*

Wilcoxon test values (*p* values) for the two-phase mutation (TPM) model. *p<0.05.

*F*_IS_ values were positive and significant (*p* = 0.05) for Hattah-Kulkyne National Park, with two loci (Smm1, Smm3) showing significant homozygote excess (Table 1). One locus (Mcym4) showed significant homozygote excess in the Murray Sunset (Central). Homozygote excess at a locus may indicate the presence of null alleles; alleles that are not expressed or their product not detected [62]. However, the detection of homozygote excess for more than one locus in the same population and without the same loci showing consistently the same pattern in other locations suggested that null alleles were probably not the cause. *F*_IS_ as a measure of inbreeding is not necessarily closely related to population or individual fitness [63], [64]. Inbreeding has several meanings depending on the reference population to which inbreeding values refer (e.g. pedigree inbreeding or homozygosity through genetic drift and low effective population size) [63], [64] and in addition, departures from Hardy-Weinberg equilibrium may be observed as a consequence of sampling strategy. Hence caution should be exercised when interpreting *F*_IS_ in the absence of other genetic metrics and demographic information. The Hattah-Kulkyne National Park population was relatively intensely sampled over the entire geographical range of this reserve, consequently this population was probably exhibiting the Wahlund effect, where a deficit of heterozygotes in a population is a result of local sub-population structure (e.g. sampling of multiple breeding groups or demes)[65]. Although the remaining Mallee Emu-wren populations showed a mixture of weak positive and negative *F*_IS_, none were significant. Linkage disequilibria were not detected for any loci pairs.

Allelic diversity (N*_A_*) was variable among loci, ranging from 2 to 32 alleles (Table 2). In general, the genetic diversity across all populations was moderate, based on allelic richness (AR, range 2.92–3.83) and expected heterozygosity (UHe, range 0.43–0.52) (Table 1). With the exception of the Murray-Sunset (West), locations were monomorphic for a number of loci that were variable elsewhere (Table 1) possibly indicating restricted local effective population size.

Two-thirds of the population pairs showed significant, but low, allele-frequency-based differentiation. Results were concordant for most pairwise comparisons of the two metrics (Table 3). Significant pairwise *F*_ST_ values were low (0.011–0.044). As was expected based on the theoretical and empirical behaviour of the metrics, significant *D_est_* values were higher than their respective *F*_ST_ values (0.077–0.179, Table 3). The Ngarkat Conservation Park population showed the greatest level of population differentiation from the other locations for both measures, which is consistent with this location being the most geographically distant, the most structurally isolated and having undergone a recent population crash (Table 3).

Significant (*p*<0.05) heterozygosity excess was detected for the Murray-Sunset National Park (East) and Ngarkat Conservation Park samples under the two-phase mutation model, indicating evidence of recent bottlenecks in these two populations (Table 4).

### Population structure

**Figure 2:**
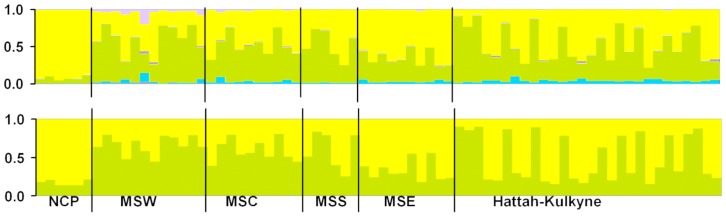
TESS boxplots for K*_max_* = 6 (top) and K*_max_* = 2 (bottom) based on 12 nuclear loci for 72 individuals. NCP = Ngarkat Conservation Park, MSW = Murray-Sunset (West), MSC = Murray-Sunset (Central), MSS = Murray-Sunset (South), MSE = Murray-Sunset (East).

**Figure 3:**
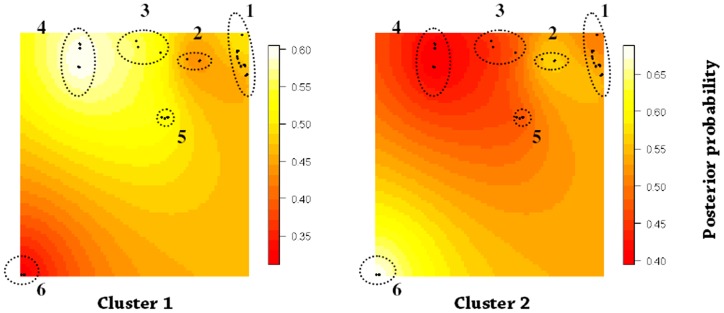
Spatially explicit predictive map of admixture coefficients as determined by TESS for 2 clusters, K*_max_* = 2. The colour scale represents the posterior probability of individuals having membership to a single genetic cluster. Black circles represent sampled individuals with known geographic locations (n = 72). Numbered ellipses indicate the populations; 1 = Hattah-Kulkyne, 2 = Murray-Sunset (East), 3 = Murray-Sunset (Central), 4 = Murray-Sunset (West), 5 = Murray-Sunset (South) and 6 = Ngarkat Conservation Park.

Overall, we found evidence of very weak population structure across the global range of the Mallee Emu-wren. STRUCTURE identified a single genetic cluster (*K* = 1). Increased power resulting from the incorporation of spatial information in TESS, revealed the presence of two weak spatial genetic clusters across the study region (Figures 2 and 3). Individuals within the geographically isolated Ngarkat Conservation Park (N^o.^ 6) were assigned to one cluster (cluster 2 in Figure 3). Excluding Murray-Sunset (East)(N^o.^ 2), the remaining populations within the Murray-Sunset and Hattah-Kulkyne reserve complex (N^os.^ 1,3,4,5) were more strongly assigned to the alternative cluster (cluster 1 in Figure 3). The distinctiveness of the Ngarkat population (for both allele frequency and genotype-based analyses) is most likely attributed to the recent population bottleneck and associated local effects of genetic drift (e.g. fixation and loss of alleles). In addition to the genetic effects of a recent population bottleneck, the isolation of the southern reserve complex, which includes Ngarkat CP, from the northern complex following clearing of vegetation for agriculture in the early 20^th^ Century [66], may represent a barrier to gene flow that has contributed to the differentiation of the Ngarkat population. There was some evidence for weak east-west structure in the Murray Sunset and Hattah Kulkyne reserve complex, probably representing contemporary, transient landscape effects or isolation-by-distance effects (Figure 3).

## Discussion

We used samples from 72 individual Mallee Emu-wrens from six separate geographical locations to examine global genetic structure and population processes in this species. Analyses of nuclear loci found low to moderate levels of genetic diversity across the species' range, and signatures of bottlenecks and local effects of genetic drift. Surprisingly, we found only weak genetic structure across the global range of the Mallee Emu-wren, contrary to expectation given its patchy distribution as a habitat specialist [20] and their widely presumed poor dispersal ability.

### Population viability

Signatures of bottlenecks in Ngarkat Conservation Park and Murry-Sunset (East) (consistent with reported demographic declines) and some evidence of genetic drift in other locations, indicate these localities may have experienced recent declines in effective population size. Bottlenecked and strongly inbreeding populations are important to identify for conservation management because of genetic threats to individual fitness and population viability [67], [68]. Small, isolated populations rapidly lose quantitative genetic variation by genetic drift. An increase in homozygosity at functional genetic diversity may lead to an increase in the expression of deleterious recessive alleles resulting in the reduction of individual fitness and inbreeding depression [18], [63], [68].

The prevalence of several monomorphic loci and evidence of a bottleneck in the Ngarkat Conservation Park population (Tables 1 & 4) is consistent with the severe contemporary decline of this population following a series of fires from 1999 to 2006 that has resulted in small, isolated groups of Mallee Emu-wrens totalling fewer than 20 pairs (C. Hedger, personal communication). These remaining groups are at immediate risk of extinction arising from stochastic environmental and demographic events and the adverse genetic affects arising from inbreeding. Although genetic information is lacking, the small number of birds detected in the Wyperfeld/Big Desert reserve complex (n = 2)[20], suggests that the persistence of the species at this location is likewise precarious. This complex also recently experienced a large fire (>180 000 ha) in 2002. With the exception of the Murray-Sunset (West), the remaining sampling locations were found to have multiple monomorphic loci, suggesting that these others may have also experienced declines in effective population size, as borne out by evidence of a bottleneck in Murray-Sunset (East). In the case of the Murray-Sunset populations, these genetic patterns may be an artefact of the low sampling intensity (<1% of the population); nevertheless, these results are consistent with proportionally greater sampling of populations in Hattah-Kulkyne NP.

Our results contrast with those from a study of the genetic effects of a forest fire on the Blue Chaffinch *Fingilla teydea polatzeki*, a critically endangered passerine endemic to the island of Gran Canaria. Despite a 50% decline in the global population of this sub-species (from about 250 to 122 individuals), temporal sampling found no genetic signature of a bottleneck. Furthermore, the post-fire population retained a high level of genetic diversity [69]. Studies directly examining the effect of fire on genetic signatures of species or populations are rare and, because of the complex nature of fire regimes, offer little in the way of direct comparison. Nevertheless, disturbance by fire has been found to reduce genetic diversity in populations of butterflies [5], [26] and has been attributed to bottlenecks in lizards [70] and anteaters [71].

A second genetic threat to the long-term viability of the Mallee Emu-wren can presumed to be the erosion of quantitative genetic variation necessary for adaptive evolution [72]. The capacity for resilience and adaptive evolution in this species is crucial because the semi-arid zone of south-eastern Australia, in which it occurs, is predicted to experience significant reduction in rainfall and more extreme temperatures with climate change [73]. There is theoretical and empirical support for the view that populations with less genetic diversity will be less able to successfully evolve with environmental change; even to the point of affecting species distributions [74]–[76]. While the relationship between neutral variation and quantitative genetic variation is not strong, population size can be a good predictor of population fitness [68], [77]. Thus, in as far as the patterns of genetic variation found here signal relatively low effective population sizes, studies of fitness would help elucidate the role that genetic variability and inbreeding may play in this species' ability to adapt to environmental change and accordingly its long-term viability.

### Landscape-scale processes

Species with limited effective dispersal are expected to show spatial genetic structure over large spatial scales. Genetic structure may be considerable, even over short distances, if the landscape matrix between habitat patches is perceived by a species to be so inhospitable as to severely limit dispersal [78]. Contrary to expectations, we found only weak genetic structure and low population differentiation among Mallee Emu-wren populations, despite this species being a very weak flier. Although genetic differentiation among several of the sampling locations was significant, the low *F*_ST_ values (0.00–0.044, Table 3) are within the range of drift connectivity (*F*_ST_≈0.1 and less); that is, populations have similar allelic frequencies indicating substantial genetic connectivity (in the order of >10 migrants per generation [79]. We note that genetic connectivity at the levels detected in this study does not preclude populations having experienced reductions in demographic connectivity.

Low population differentiation does not necessarily imply contemporary genetic connectivity; for example, recently isolated populations or populations with large effective population size could show population differentiation in the absence of connectivity because of the time lag before the genetic consequences of fragmentation and isolation become manifest [50]. However, this is not likely to be the case with the Mallee Emu-wren: subpopulations are demographically small and most of the species' distribution is within an expansive intact landscape. High genetic connectivity across fragmented landscapes has been demonstrated for other bird species with low mobility, including the closely allied Superb Fairy-wren[80], [81]. This latter species showed large-scale gene flow, but even so, landscape change can still have adverse consequences for fine-scale population processes such as mating systems and song sharing [80]–[82].

Collectively, the genetic patterns and population structure found in this study can be attributed to the spatial and temporal patterns of fire in mallee ecosystems. Most fires are small (<100 ha in size), but intense landscape-scale wildfires exceeding 10,000 ha occur within the region every 10–20 years [83], [84]. The spatial distribution of residual survivors is crucial to understanding the process of population recovery and its genetic consequences at a local scale after fire. Population recovery may occur either by recolonisation by individuals originating beyond the boundary of the fire footprint, or there may be residual survivors within unburnt refuges enabling *in-situ* recovery (i.e. nucleated recovery) from within the fire footprint [9], [16], [27]. These contrasting processes could lead to different genetic signatures in recovering populations. Extirpation of populations and recolonisation by founders mostly (but not invariably) leads to population bottlenecks, founder effects, enhanced effects of drift and the erosion of genetic diversity [85], [86]. In contrast, population recovery from *in-situ* survivors is less likely to be accompanied by loss of much original genetic diversity, except in the presence of very strong, sustained or repeated bottlenecks [87]. In actuality, these two processes (recovery based on immigrants vs. nucleated recovery) are not mutually exclusive but more likely the two extremes of a continuum.

Given large, severe wildfires dominate the mallee landscape [88], it is likely that recolonisation exceeds *in-situ* survival as the primary means of population recovery of the Mallee Emu-wren. These large fires (>10,000 ha) create vast homogenous areas in which the ground layer (including fallen timber), shrub and canopy strata are all consumed [89]. Denuded of vegetation, the burnt landscape is unable to support (even temporarily) species such as the Mallee Emu-wren that depend on mid to late seral-stage vegetation. Serial founder and recolonisation events resulting from such fires, have most likely eroded genetic variability in this species. Recolonisation as a primary driver of population recovery is consistent with findings of a contemporary study on birds in mallee ecosystems [27] and for birds in fire-prone Mediterranean ecosystems of Europe [90]. Recolonisation may also drive population processes in other species with low mobility and dependent on ground-cover dependent that inhabit fire-prone landscapes, such as the Grasswrens *Amytornis spp*. of the arid-zones of Australia [29], and the small marsupial, the mallee Ningaui *Ningaui yvonneae* [91].

In contrast, recovery from *in-situ* survival may occur in environments where fires leave numerous unburnt refuges, as in the case of the Blue Chaffinch of Gran Canaria discussed earlier. Unburnt refuges were prevalent throughout the fire area and were thought to enable a sufficient proportion of individuals to survive and persist, thereby mitigating the loss of genetic variability in the post-bottleneck population [69]. *In-situ* survivorship in unburnt refuges has been attributed to the rapid demographic recovery in birds (e.g. the fire-sensitive Eastern Bristlebird *Dasyornis bachypterus* [92]) and the persistence of species diversity and genetic diversity in invertebrates following large fires [5], [26], [93].

Extinction and recolonisation in fire-prone landscapes can also lead to increased genetic variance and differentiation among some populations [11], [70], but this does not appear to be the situation for the Mallee Emu-wren. Rather we propose that the shifting patch mosaic characteristic of mallee shrublands facilitates genetic connectivity for the Mallee Emu-wren as sub-populations spatially track suitable successional vegetation (habitat-tracking). In mallee shrublands, fire is a stand-replacing disturbance, where vegetation succession is very gradual, peaking in structural complexity at about 30 years of age. Vegetation also may remain unburnt for over a century [89]. Where specialist species such as the Mallee Emu-wren have life-history traits that limit dispersal, successional patch dynamics will facilitate movement, and hence gene flow, across the wider landscape. Such gene flow and genetic structure of sub-populations will be influenced by the rate of the shifting habitat mosaic. Accumulation of genetic differentiation in populations of the Mallee Emu-wren may be only transient as movement among temporally and spatially shifting habitat, mediated by fire, occurs on a timescale faster than new variation arises, thus acting to homogenise genetic structure in this species.

### Implications for conservation

The apparent genetic connectivity of the Mallee Emu-wren inferred from the low population differentiation (F_ST_), and weak genetic structure (STRUCTURE/TESS) is an optimistic message for the conservation of this species. The lack of marked population differentiation across its global range means that for management purposes (e.g. translocation of individuals) this species can be treated as a single genetic unit. Nevertheless, the finding of disrupted fine-scale population processes, as illustrated by the demographic and genetic impoverishment in the Ngarkat Conservation Park population, supports implementation of actions to assist population recovery. Reintroduction programs or the genetic restoration [72]of the Ngarkat population can be undertaken with minimal genetic risk from outbreeding depression, which for species of conservation concern is generally outweighed by inbreeding depression [94]. That said, other, non-genetic factors including disease and the demographic impacts of management interventions also need to be considered [95], [96].

Prescribed burning is a tool widely used in fire-prone ecosystems to reduce the risk to life, to protect ecological and built assets, and to prevent landscape-scale fires burning extensive areas and homogenising the landscape [97]. Rare, fire-sensitive species with low mobility or which are site tenacious, such the Black-eared Miner [98], the Eastern Bristlebird [92] and the Mallee Emu-wren, will benefit from approaches to fire management that prevents large-scale fires. Whilst providing for this broad goal however, it is imperative that prescribed burns are of appropriate size and spatio-temporal arrangement (fire mosaic) so as not to disrupt movement between suitable patches of habitat, allowing for gene flow among sub-populations. A second aspect to consider in the development of fire management plans is the importance of refuges. *In-situ* residual populations from unburnt refuges can enhance subsequent recovery to post-fire areas by providing individuals for population growth [27], [90], [93]. These immigrants may also help mitigate the erosion of genetic diversity and homogenisation within founder populations by contributing new alleles to the gene pool. Hence, maintaining unburnt patches with key habitat attributes for specialist species is an appropriate objective for fire management.

## Supporting Information

Table S1 Parent-offspring pairs identified by CERVUS parentage analysis.
(DOCX)

Text S1 Summary of CERVUS parentage analysis.
(DOCX)
