# Supplementary material for: Limited Population Structure, Genetic Drift and Bottlenecks Characterise an Endangered Bird Species in a Dynamic, Fire-Prone Ecosystem
Source: PLoS One. 2013 Apr 23;8(4):e59732. doi: 10.1371/journal.pone.0059732 (PMC3634030; doi:10.1371/journal.pone.0059732)
Supplement: Table S1 — Parent-offspring pairs identified by CERVUS parentage analysis. (DOCX) [file pone.0059732.s001.docx]

**Table S1.**

Parent-offspring pairs identified by CERVUS parentage analysis. Table lists individual identification (Offspring ID), sampling location and sex (M=male, F=female) for offspring and assigned parent under relaxed 80% (+) and strict 95% (*) confidence. Individuals removed from analyses are highlighted in bold font.

| **Offspring ID** | **Sampling location** | **Offspring sex** | **Assigned parent ID** | **Sampling location** | **Parent sex** | **Confidence** |
| --- | --- | --- | --- | --- | --- | --- |
| **15HAT** | 002 | M | 14HAT | 002 | F | * |
| 27HAT | Shingleback Tk | F | 45PL | Lk Crosbie Walking Tk | F | * |
| 41MSW | Sth Bore Tk | F | 75NAR | NGARKAT | F | * |
| 45MSS | Lk Crosbie Walking Tk | F | 27HAT | Shingleback Tk | F | * |
| 65MSE | Last Hp Tk East | F | 66MSE | Last Hp Tk East | F | * |
| 66MSE | Last Hp Tk East | F | 65MSE | Last Hp Tk East | F | * |
| 72NAR | NGARKAT | M | 74NAR | NGARKAT | F | * |
| **17HAT** | 003 | M | 20HAT | Nowingi Tk, near cp | F | + |
| 48PLK | Pink Lk | M | 47MSS | Pink Lk | M | + |
| 67MSE | Last Hope Tk West | F | 70MSE | Last Hope Tk West | F | + |
| 70MSE | Last Hope Tk West | F | 67MSE | Last Hope Tk West | F | + |
| 76NAR | NGARKAT | F | 75NAR | NGARKAT | F | + |
| **87HAT** | SS, Nest 01 | chick | 30HAT | SS, Nest 01 | F | + |
| 14HAT | 002 | F | **15HAT** | 002 | M | * |
| **22HAT** | Compound Gate | M | 29HAT | Far back compound | M | * |
| 29HAT | Far back compound | M | **22HAT** | Compound Gate | M | * |
| 47MSS | Pink Lk | M | 48MSS | Pink Lk | M | * |
| 74NAR | NGARKAT | F | 72NAR | NGARKAT | M | * |
| **13HAT** | Nowingi East side of A gully | M | 03HAT | Nowingi Site B east | M | + |
| 20HAT | Nowingi Tk, near cp | F | **17HAT** | 003 | M | + |
| 24HAT | near 008 | M | 42MSW | Sth Bore Tk | M | + |
| 32MSW | MS SthBore Tk No 5 | M | 33MSW | MS SthBore Tk No 5 | M | + |
| 33MSW | MS SthBore Tk No 5 | M | 32MSW | MS SthBore Tk No 5 | M | + |
| 69MSE | Last Hope Tk West | M | 68MSE | Last Hope Tk West | M | + |
| 79HAT | Sth MV hwy | F | **80HAT** | Sth MV hwy | M | + |
