# Supplementary material for: Limited Population Structure, Genetic Drift and Bottlenecks Characterise an Endangered Bird Species in a Dynamic, Fire-Prone Ecosystem
Source: PLoS One. 2013 Apr 23;8(4):e59732. doi: 10.1371/journal.pone.0059732 (PMC3634030; doi:10.1371/journal.pone.0059732)
Supplement: Text S1 — Summary of CERVUS parentage analysis. (DOCX) [file pone.0059732.s002.docx]

**Text S1.**

**Summary of CERVUS parentage analysis**

Parentage analysis was performed in CERVUS 3.0 [34]. Simulations were carried out with 10,000 offspring and the proportion of 40,000 candidate mothers/fathers sampled was set to 0.25. We used a conservative estimate of 200 potential parents for each bird sampled. The proportion of successfully genotyped loci was 98.7% (based on our data), with a genotyping error rate of 1%. Parentage analysis was performed using parent-pair with sexes known. For each offspring, candidate parents were assigned a Delta score, and the confidence of assignment of the most-likely candidate parent (highest Delta score) was assessed at two levels of statistical confidence (relaxed 80% and strict 95%).

**Results**

Under relaxed (80%) confidence of parentage assignment, CERVUS analysis identified 16 potential parent-offspring pairs. We preferentially removed males (the more philopatric sex) rather than females and, given already very low samples, chose to remove potential offspring of sampled parents from only the most densely sampled Hattah-Kulkyne National Park population. Six potential offspring originating from the Hattah-Kulkyne population were removed from the dataset (Table S1). Of the remaining 10 pairs, 4 were improbable parent-offspring pairs because of the large geographical distances of origin (5 - >100 km) and were therefore retained in the dataset (i.e. 72NAR/74NAR, 41MSW/75NAR, 27HAT/45MSS, 24HAT/42MSW, Table S1). Only a single parent-offspring pair (65MSE and 66MSE) was identified with strict confidence (Table S1), so sampling of parent-offspring pairs is unlikely to strongly bias the genetic signatures at the remaining five locations. Two additional chicks sampled at the same nest as 87HAT were removed from analyses.
